# The complete chloroplast genome of *Cymbidium hookerianum* (Orchidaceae): genome structure and basic analysis

**DOI:** 10.1080/23802359.2020.1845996

**Published:** 2021-01-05

**Authors:** Ziling Wei, Bin Chen, Yinghui Cao, Yan Zheng, Yanping Zhang, Kai Zhao, Yuzhen Zhou

**Affiliations:** a Ornamental Plant Germplasm Resources Innovation and Engineering Application Research Center at College of Landscape Architecture, Key Laboratory of National Forestry and Grassland Administration for Orchid Conservation and Utilization, College of Landscape Architecture, Fujian Agriculture and Forestry University, Fuzhou, P. R. China; b College of Life Sciences, Fujian Normal University, Fuzhou, China

**Keywords:** *Cymbidium hookerianum*, chloroplast genome, phylogenetics

## Abstract

*Cymbidium hookerianum* Rchb.f. is an ornamental orchid with large flowers and delicate aroma. Here, we reported the complete chloroplast genome sequence of *C. hookerianum*. The total chloroplast genome cycle was 155,447 bp. It displayed a typical structure including one large single-copy (LSC, 84,186 bp) region, one small single-copy (SSC, 17,839 bp) region, and two inverted repeat (IRs, 26,711 bp). 124 genes (78 CDSs, 38 tRNAs, 8 rRNAs) were encoded by the cp genome. The average GC content of this sequence was 36.8%. The phylogenetic analysis revealed *C. hookerianum* and *C. changningense* are sisters. The groundwork of chloroplast genome would provide available reference for molecular taxonomy and breeding.

*Orchidaceae* is the largest family in monocotyledons. As a member of the Subgen *Cyperorchis* in *Orchidaceae*, *Cymbidium hookerianum* Rchb.f. is characterized by large flowers and slight aroma (Liu et al. 2009). *Cymbidium hookerianum* enjoys a shady and moist forest environment at an altitude of 1500–2600 m in northern Yunnan, eastern Nepal and north-eastern India (DuPuy et al. 2007). It is usually hybridized under natural conditions. Simultaneously, owing to the fascinating floral characteristics, this species has been selected as superior parents and bred hundreds of cultivars in intraspecific and interspecific (DuPuy et al. 1985). What is more, *Cymbidum* hybrids, cultivated by *C. hookerianum*, has become a popular production for trade worldwide. However, there are many kinds of natural and cultivated hybrids which brings a lot of difficulties in the identification and selection of parents. And most of the classification of Cymbidium is built on morphology (Yang et al. 2013). As for *C. hookerianum*, no complete genome has been reported. Now, the complete chloroplast genome helps to reveal the genetic taxonomy at molecular level, and serves as a systematic understanding of the status of this species in the genus. Also, it displays a clear guideline for breeding new cultivars.

Leaf samples of *C. hookerianum* were acquired from Fuzhou, Fujian province, China, and preserved in Fujian Agriculture and Forestry University (Voucher specimen: HTL-FJ2019-28A, 26°20′21.3″ N, 113°12′39.6″ E). Total genomic DNA was extracted from fresh leaves by modified CTAB procedure, and sequenced on the BGI-500 platform (BGI, Wuhan, China) (Mak et al. 2017). The reads length of raw sequencing data is 150 bp. About 10 Gb clean reads were obtained after moving adapters and unreliable reads by FASTQ software (Chen et al. 2018). Filtered data was assembled by bowtie2 and Spades (k-mer parameters: -k 21,45,65,85,105). Then, geneious prime 2020.2.1and Dual Organellar GenoMe Annotator (DOGMA) (Wyman et al. 2004) and MEGAX version10.1.8 (Kumar et al. 2018) were tools to accomplish annotation.

A complete circular chloroplast sequence of *C. hookerianum*(MT800927), with a total length of 1,55,447 bp, was established to include one large single copy (LSC, 84,186 bp) region, one small single copy (SSC,17,839 bp) region, and two inverted repeat (IRs, 26,711 bp) regions. The average GC content of this sequence was 36.8%, and every region had uneven GC content: LSC: 34.3%, SSC: 29.5%, IRs: 43.1%, respectively. It had been found that 124 genes (78 CDSs, 38 tRNAs, 8 rRNAs) were encoded in the cp genome.

To investigate the phylogenetic relationships of *C. hookerianum*, the complete chloroplast sequence of 24 species, among which 22 sequences are *Cymbidium* and the other two are outgroups, was illustrated as the phylogenetic dendrogram. These sequences were aligned in geneious prime 2020.2.1 by Mafft Multiple Alignment plugin version 1.4.0, and a maximum-likelihood tree (ML) with 1000 bootstrap replicates was built by RAxML-HPC2 program (Stamatakis et al. 2008) on CIPRES (Miller et al. 2010). The dendrogram showed that *C. hookerianum* was more closely related to *C. changningense* and formed a clade with other 6 species from Sect. Iridorchis and Sect. *Eburnea*. Among the 6 species, *C. tracyanum, C. erythraeum, and C. hookerianum* are in the Sect. *Iridorchis* of *Cymbidium* (DuPuy et al. 1985). However, we are interested to notice that *C. tracyanum* has a further kinship with other members of Sect. *Iridorchis* than with Sect. *Eburnea* (Figure 1). We believe this research gives a more valid molecular taxon evidence, and supplies effective genomic resources for *Cymbidium* hybridizing.

**Figure 1.**
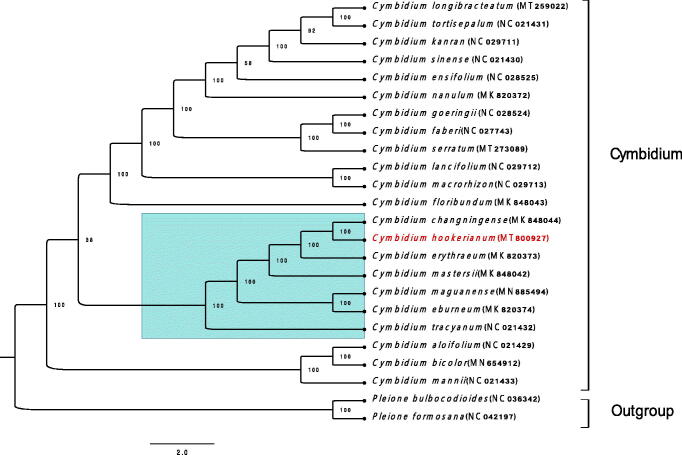
The maximum-likelihood (ML) phylogenetic tree of Cymbidium was founded on 24 selected complete chloroplast genomes. *Cymbidium hookerianum* was marked in bold and red. All sequence data were downloaded from GenBank.

## Data Availability

The *Cymbidium hookerianum* raw data has been stored in omics database of Genome Sequence Archive. GSA accession number is CRA003135. All the information can be found on the website (https://bigd.big.ac.cn/gsa/browse/CRA003135/CRX151933). The data that support the findings will be available in GenBank at (https://www.ncbi.nlm.nih.gov/nuccore/MT800927).
